# The risk of skin cancer in women who carry *BRCA1* or *BRCA2* mutations

**DOI:** 10.1186/s13053-024-00277-5

**Published:** 2024-05-13

**Authors:** Steven A. Narod, Kelly Metcalfe, Amy Finch, An-Wen Chan, Susan Randall Armel, Amber Aeilts, Andrea Eisen, Beth Karlan, Louise Bordeleau, Nadine Tung, William D. Foulkes, Susan L. Neuhausen, Charis Eng, Olufunmilayo Olopade, Dana Zakalik, Fergus Couch, Carey Cullinane, Tuya Pal, Ping Sun, Joanne Kotsopoulos, the Hereditary Breast Cancer Clinical Research Group, Aletta Poll, Raymond Kim, Robert Fruscio, Edmond Lemire, Kim Serfas, Kevin Sweet, Leigha Senter, Seema Panchal, Christine Elser, Joanne L. Blum, Daniel Rayson, Claudine Isaacs, Jeffrey Dungan, Stephanie Cohen

**Affiliations:** 1 Women’s College Research Institute, Women’s College Hospital, 76 Grenville St, M5S 1B1 Toronto, ON Canada; 2 Dalla Lana School of Public Health, University of Toronto, Toronto, ON Canada; 3 Bloomberg School of Nursing, University of Toronto, Toronto, ON Canada; 4 Division of Dermatology, Department of Medicine, University of Toronto, Toronto, Canada; 5 Princess Margaret Hospital, Familial Cancer Clinic, University Health Network, Toronto, ON Canada; 6 Department of Molecular Genetics, University of Toronto, Toronto, ON Canada; 7 Division of Human Genetics, Comprehensive Cancer Center, the Ohio State University Medical Center, Columbus, OH USA; 8 Odette Cancer Center, Toronto, ON Canada; 9 Department of Obstetrics and Gynecology, David Geffen School of Medicine, University of California, Los Angeles, CA USA; 10 Department of Oncology, McMaster University, Hamilton, ON Canada; 11 Beth Israel Deaconess Medical Center, Boston, MA USA; 12 Program in Cancer Genetics, Department of Oncology and Human Genetics, McGill University, Montréal, QC Canada; 13 Department of Population Sciences, Beckman Research Institute of City of Hope, Duarte, CA USA; 14 Genomic Medicine Institute, Cleveland Clinic, Cleveland, OH USA; 15 Department of Medicine and Human Genetics, University of Chicago, Chicago, IL USA; 16 Cancer Genetics Program, Beaumont Hospital, Royal Oak, MI USA; 17 Division of Experimental Pathology and Laboratory Medicine, Department of Laboratory Medicine and Pathology, Mayo Clinic, Rochester, MN USA; 18 MemorialCare Long Beach Medical Center, Long Beach, USA; 19 Division of Genetics, Department of Medicine, Vanderbilt University Medical Centre and Vanderbilt-Ingram Cancer Center, Nashville, TN USA

**Keywords:** Skin cancer, Basal cell carcinoma, Melanoma, BRCA1, BRCA2

## Abstract

**Background:**

It has not been clearly established if skin cancer or melanoma are manifestations of *BRCA1* or *BRCA2* mutation carrier status. Estimating the risk of skin cancer is an important step towards developing screening recommendations.

**Methods:**

We report the findings of a prospective cohort study of 6,207 women from North America who carry *BRCA1* or *BRCA2* mutations. Women were followed from the date of baseline questionnaire to the diagnosis of skin cancer, to age 80 years, death from any cause, or the date of last follow-up.

**Results:**

During the mean follow-up period of eight years, 3.7% of women with a *BRCA1* mutation (133 of 3,623) and 3.8% of women with a *BRCA2* mutation (99 of 2,584) reported a diagnosis of skin cancer (including both keratinocyte carcinomas and melanoma). The cumulative risk of all types of skin cancer from age 20 to 80 years was 14.1% for *BRCA1* carriers and 10.7% for *BRCA2* carriers. The cumulative risk of melanoma was 2.5% for *BRCA1* carriers and 2.3% for *BRCA2* carriers, compared to 1.5% for women in the general population in the United States. The strongest risk factor for skin cancer was a prior diagnosis of skin cancer.

**Conclusion:**

The risk of non-melanoma skin cancer in women who carry a mutation in *BRCA1* or *BRCA2* is similar to that of non-carrier women. The risk of melanoma appears to be slightly elevated. We suggest that a referral to a dermatologist or primary care provider for *BRCA* mutation carriers for annual skin examination and counselling regarding limiting UV exposure, the use of sunscreen and recognizing the early signs of melanoma might be warranted, but further studies are necessary.

## Introduction

The major categories of skin cancer are malignant melanoma and non-melanoma skin cancers (keratinocyte carcinoma) which include basal cell carcinomas and squamous cell carcinomas [1]. The World Health Organization estimates that 1,200,000 cases of non-melanoma skin cancer and 325,000 cases of melanoma were diagnosed in 2020 (with 64,000 deaths and 57,000 deaths respectively) [2].

Risk factors for skin cancer include sun exposure (ultraviolet light), fair skin and eye colour, and a family history of skin cancer [3]. Germline mutations in several genes have also been associated with skin cancer predisposition. Variants in *SUFU* and *PTCH* are responsible for the nevoid basal cell carcinoma syndrome (NBCCS) also known as Gorlin syndrome [4, 5]. Variants in *CDKN2A* are responsible for Familial Atypical Multiple Mole Melanoma syndrome (FAMMM), which accounts for almost 40% of families with multiple cases of cutaneous melanoma [6, 7]. Other genes associated with the development of melanoma include *PTEN, CDK4, ACD, TERF2IP BAP1, POT1, MITF, MC1R, TP53, RB1 and TERT* [8–15]. Together, the known predisposition genes account for approximately 50% of familial melanoma cases [9] but fewer than 10% of all melanoma cases.

Women who carry a *BRCA1* or *BRCA2* mutation have elevated risks for many types of cancer, including breast, ovarian, fallopian tube and peritoneal cancers [16]. Men are at increased risk for prostate cancer and both sexes are at risk for pancreatic cancer. The evidence for an increased risk of skin cancer is inconsistent. Early studies of women who carry *BRCA* mutations found an increased risk of melanoma in family members of *BRCA2* carriers [17, 18] However, this was not seen in a recent study of *BRCA1* and *BRCA2* families described by the CIMBA consortium [19]. Family based studies may be subject to bias and all relatives are not known to be carriers. A better approach is to follow a cohort of *BRCA* carriers over time and document new cases of skin cancer in established carriers. We sought to estimate the annual and lifetime risks for skin cancer associated with *BRCA* mutations in order to determine if screening measures should be adopted.

## Methods

Study participants were identified from a multi-centre longitudinal study of women who carry *BRCA1* or *BRCA2* mutations (pathogenic variants). Women were drawn from participants in ongoing studies at 52 participating centres throughout Canada and the United States. All centres received approval by their Research Ethics Board or Institutional Review Board. Each participant provided informed consent and filled out a baseline questionnaire at study entry and a follow-up questionnaire every two years thereafter. These questionnaires included information about personal history of cancer including skin cancer, prior cancer diagnoses, family history of cancer, and ethnicity Women were included if they had completed at least one follow-up questionnaire and were excluded if they were lost to follow-up or were missing *BRCA* mutation status. Women with a previous diagnosis of cancer, including breast, ovarian, skin or other cancer at study entry were included. Family history of skin cancer in a first-degree relative was recorded when available. The majority of the participants were white of European ancestry.

Participants were asked in each follow-up questionnaire if they had been diagnosed with any cancer using an open-ended question. Skin cancer diagnoses were based on patient report and fell into three categories: basal cell carcinoma, melanoma or ‘skin cancer’. Women were followed from the date of their baseline questionnaire until diagnosis of skin cancer, date of last follow-up questionnaire or death.

### Statistical analysis

The annual risks for skin cancer (all types) and for melanoma were estimated by age group (20–29, 30–39, 40–49, 50–59, 60–69, and 70–79 years) for *BRCA1* and *BRCA2* carriers by calculating the ratio of number of events (cancer diagnoses) and the number of person years in each group. Person years were accumulated in the cohort for each age group (e.g. age 20–29 years) for those women who were alive and cancer-free at the beginning of each age group (e.g. age 20 years). The number of skin cancers that developed in women in that age group were then counted.

The cumulative incidence of skin cancer (any) and of melanoma was calculated using the Kaplan-Meier method. Patients were followed from age 20 or baseline (whichever came last) until the diagnosis of skin cancer, death from any cause, age 80 or the date of the last follow up.

The univariate and multivariate hazard ratios (HR) and 95% confidence intervals (CIs) associated with various risk factors for skin cancer were estimated using the Cox Proportional Hazards model. Exposures of interest included a family history of skin cancer, a personal history of skin cancer, past history of breast cancer and hormone replacement therapy. Subgroup analysis was performed by mutation status (*BRCA1, BRCA2*) and by type of skin cancer (all skin cancer, melanoma). All analyses were conducted using the SAS statistical package, version 9.4 software (SAS Institute, Cary, NC, USA). All P values were two-sided and were considered statistically significant if *p* < 0.05.

## Results

A total of 6,207 women were included in the analysis: 3,623 women with *BRCA1* mutations and 2,594 women with *BRCA2* mutations (see Table 1). The average duration of follow-up from baseline was eight years. A previous diagnosis of breast cancer was reported by 45.5% of participants and a previous diagnosis of ovarian cancer was reported by 10.5%. A previous diagnosis of skin cancer was reported by 3.4% of *BRCA1* mutation carriers and by 4.1% of *BRCA2* mutation carriers. 173 women reported a family history of skin cancer in a first-degree relative (6.7%).

**Table 1:** Characteristics of the 6207 subjects

	Mean (range) or Frequency (%)
Date of birth (year)	1958 (1908–1997)
Date of baseline (year)	2005 (1994–2020)
Years of follow-up	8.1 (0.1–25.1)
**Ethnic group**	
Jewish	1205
French-Canadian	520
Other white	4153
African-American	90
Asian	129
Other	110
*BRCA1*	3623 (58.4%)
*BRCA2*	2584 (41.6%)
**Country of residence**	
USA	3271 (52.7)
Canada	2936 (47.3)
Breast cancer at baseline	
No	3381 (54.5%)
Yes	2819 (45.5%)
Missing	7
**Ovarian cancer at baseline**	
No	5456 (87.9)
Yes	653 (10.5)
Missing	98
**Hormone replacement therapy**	
No	4024 (64.9)
Yes	2180 (35.1)
Missing	3
**Oophorectomy**	
No	1977 (32.0)
Yes	4206 (68.0)
Missing yes/no	24
Mean year of oophorectomy	2003.6 (1945–2021)
Mean age of oophorectomy	46.9 (13–83)
**Skin cancer at baseline**	
No	5952 (95.5)
Yes	255 (4.1)
**First degree relative with skin cancer**	
No	2403 (93.3)
Yes	173 (6.7)
Missing	3631
**Skin cancer at follow-up**	
Any	232 (3.7%)
Basal cell carcinoma	88 (1.4%)
Melanoma	44 (0.7%
‘Skin’	100 (1.6%)

During the follow-up period, 232 women reported a diagnosis of skin cancer (3.7%); 88 basal cell carcinomas, 44 melanomas and 100 ‘skin cancers’. The age-specific annual risks for skin cancer (all types combined) and for melanoma are presented in Tables 2a and 2b. Overall, between age 40 and 79 the annual risk for any skin cancer was 0.5% for *BRCA1* carriers and 0.6% for *BRCA2* carriers. Between age 40 and 79 the annual risk for melanoma was 0.09% for *BRCA1* carriers (90/100,000 per year) and 0.12% for *BRCA2* carriers (120 per 100,000 per year). We estimated the cumulative risk of any skin cancer from age 20 to 80 to be 14.1% for *BRCA1* mutation carriers and 10.7% for *BRCA2* mutation carriers. We estimated the cumulative risk of melanoma between age 20 and 80 years to be 2.5% for women who carry *BRCA1* mutations and 2.3% for women who carry *BRCA2* mutations. Women from the United States had a higher risk of skin cancer than those from Canada (HR = 1.98, 95% CI: 1.39, 2.83, *p* < 0.0001) for *BRCA1* carriers and HR = 1.55 (95%CI 1.04–2.30; *p* = 0.03) for *BRCA2* carriers (Tables3a and 3b).

**Table 2a:** Annual risks for skin cancer (all types) by age and gene

	BRCA1			BRCA2		
Age group	Number of person years	Number of events	Annual risk (%)	Number of person years	Number of events	Annual risk (%)
20–29	1166	0	0	559	0	0
30–39	4218	8	0.2%	2331	4	0.2%
40–49	8318	30	0.4%	4652	21	0.5%
50–59	8594	49	0.6%	5985	37	0.6%
60–69	5174	23	0.4%	4316	25	0.6%
70–79	2042	23	1.1%	1910	10	0.5%

**Table 2b:** Annual risks for melanoma by age and gene

	BRCA1			BRCA2		
Age group	Number of person years	Number of events	Annual risk (%)	Number of person years	Number of events	Annual risk (%)
20–29	1166	0	0.0%	559	0	0
30–39	4218	1	0.02%	2331	1	0.04%
40–49	8318	7	0.08%	4652	4	0.09%
50–59	8594	8	0.09%	5985	11	0.18%
60–69	5174	4	0.08%	4316	2	0.05%
70–79	2042	3	0.15%	1910	3	0.16%

**Table 3a:** Hazard ratios for skin cancer for some variables (*BRCA1* subjects)

Variables	Controls/ cases	Univariate HR (95%CI) P-value	Multivariate HR (95%CI) P-value
Age at baseline in years			
<=40	1208/34	1	1
41–50	1065/44	1.51 (0.96, 2.36) 0.07	1.22 (0.76, 1.96) 0.40
>=51	1217/55	1.86 (1.21, 2.85) 0.005	1.50 (0.94, 2.41) 0.09
Trend by year of age		1.03 (1.01, 1.04) 0.0003	1.02 (1.02, 1.04) 0.01
Breast cancer at baseline			
No	1922/57	1	1
Yes	1566/76	1.77 ( 1.26, 2.50) 0.001	1.45 (1.00, 2.11) 0.05
Ovarian cancer at baseline			
No	2989/125	1	1
Yes	444/8	0.63 (0.31, 1.29) 0.21	0.84 (0.37, 1.91) 0.67
Hormone replacement therapy			
No	2231/80	1	1
Yes	1253/58	0.93 (0.66, 1.33) 0.70	0.88 (0.61, 1.28) 0.51
Skin cancer at baseline			
No	3367/118	1	1
Yes	123/15	3.84 (2.24, 6.58) < 0.0001	2.77 (1.60, 4.82) 0.0003
Family history of skin cancer			
No	1345/74	1	1
Yes	90/10	1.99 (1.03, 3.84) 0.04	1.85 (0.95, 3.13) 0.07
Country			
Canada	1569/48	1	1
USA	1921/85	1.98 (1.39, 2.83) 0.0002	1.70 (0.93–3.09) 0.08

**Table 3b:** Hazard ratios for skin cancer for some variables (*BRCA2* subjects)

Variables	Controls/ cases	Univariate HR (95%CI) P-value	Multivariate HR (95%CI) P-value
Age at baseline in years			
<=40	685/18	1	1
41–50	724/31	1.53 (0.85, 2.73) 0.15	1.46 (0.79, 2.71) 0.23
>=51	1076/50	1.89 (1.10, 3.24) 0.02	1.79 (0.98, 3.27) 0.06
Trend by year of age		1.02 (1.00, 1.03) 0.03	1.02 (1.00, 1.04) 0.05
Breast cancer at baseline			
No	1349/53	1	1
Yes	1131/46	1.13(0.76, 1.67) 0.56	1.03 (0.67, 1.57) 0.90
Ovarian cancer at baseline			
No	2249/93	1	1
Yes	195/6	0.95 (0.42, 2.18) 0.91	0.79 (0.31-2.01 )0.62
Hormone replacement therapy			
No	1655/58	1	1
Yes	828/41	1.29 (0.87, 1.93) 0.21	1.06 (0.69, 1.62) 0.80
Skin cancer at baseline			
No	2379/88	1	1
Yes	106/11	2.80 (1.50, 5.24) 0.001	2.17 (1.13, 4.15) 0.02
Family history of skin cancer			
No	927/57	1	1
Yes	65/8	1.71 (0.81,3.57) 0.16	1.41 (0.67, 2.99) 0.37
Country			
Canada	1269/50	1	1
USA	1216/49	1.55 (1.04, 2.30) 0.03	1.55 (0.82-2.94) 0.18

A diagnosis of skin cancer prior to study entry was the strongest risk factor for development of skin cancer (all types) (Tables 3a and 3b). The univariate hazard ratio was similar for *BRCA1* carriers (HR = 3.84, 95% CI: 2.24–6.58, *p* < 0.001) and for *BRCA2* carriers (HR = 2.80, 95% CI: 1.50–5.24, *p* = 0.001).

Overall, four of the 44 women with a new diagnosis of melanoma had a prior diagnosis of melanoma. Women who reported a diagnosis of melanoma prior to study entry had a significantly elevated risk for a new diagnosis of melanoma (Tables 4a and 4b). The hazard ratio was 11.9 (95% CI: 2.78–50.9; *p* = 0.0008) for *BRCA1* carriers and was 9.90 (95% CI 2.28–4.27; *p* = 0.002) for *BRCA2* carriers (see Table 4a and 4b). None of the women with incident melanoma had a family history of melanoma.

**Table 4a:** Hazard ratios for melanoma for some variables (*BRCA1* subjects)

Variables	Controls/ cases	Univariate HR (95%CI) P-value	Multivariate HR (95%CI) P-value
Age at baseline in years			
<=40	1237/5	1	1
41–50	1099/10	2.40 (0.82, 7.03) 0.11	1.90(0.63, 5.73) 0.26
>=51	1264/8	1.80 (0.59, 5.51) 0.30	1.47(0.46, 4.73) 0.52
Breast cancer at baseline			
No	1969/10	1	1
Yes	1629/13	1.72 (0.76, 3.93) 0.20	1.61 (0.66, 3.92) 0.30
Hormone replacement therapy			
No	2300/11	1	1
Yes	1299/12	1.65 (0.73, 3.74) 0.23	1.62 (0.68, 3.85) 0.28
Melanoma at baseline			
No	3481/20	1	1
Yes	28/2	11.9 (2.78, 50.9) 0.0008	9.45 (2.16, 41.3) 0.003
Family history of melanoma			
No	1406/13	1	1
Yes, melanoma	9/0	0	0
Country			
Canada	1610/7	1	1
USA	1990/16	2.41 (1.00, 5.93) 0.05	1.26 (0.35–4.58) 0.73

**Table 4b:** Hazard ratios for melanoma for some variables (*BRCA2* subjects)

Variables	Controls/cases	Univariate HR (95%CI) P-value	Multivariate HR (95%CI) P-value
Age at baseline in years			
<=40	698/5	1	1
41–50	749/6	1.08 (0.33, 3.55) 0.89	1.10 (0.31, 3.88) 0.88
>=51	1116/10	1.38 (0.47, 4.08) 0.56	1.56 (0.48, 5.09) 0.46
Breast cancer			
No	1388/14	1	1
Yes	1170/7	0.65 (0.26, 1.61) 0.35	0.69 (0.26, 1.81) 0.44
Hormone replacement therapy			
No	1702/11	1	1
Yes	859/10	1.66 (0.70, 3.90) 0.25	1.22 (0.48, 3.07) 0.68
Melanoma at baseline			
No	2449/18	1	1
Yes, melanoma	27/2	9.90 (2.28, 42.7) 0.002	10.9 (2.41, 49.1) 0.002
Country			
Canada	1303/16	1	1
USA	1260/5	0.46 (0.17, 1.26) 0.13	0.40 (0.07–2.21) 0.29

## Discussion

We estimated the annual and cumulative risk of skin cancer in women who carry *BRCA1* or *BRCA2* mutations. We found the risk for melanoma in *BRCA1* and *BRCA2* mutation carriers to be only slightly higher than expected, based on US population rates. The lifetime risk of melanoma was approximately 2.5% for women who carried *BRCA1* mutations and 2.1% for women who carried *BRCA2* mutations, compared to 1.5% for white women in the general population in the United States [20]. Similar data are not available for non-melanoma skin cancer. Moran et al. reported two cases of uveal melanoma in 222 families with *BRCA2* mutations (HR = 99.4; 95%:CI 11.1–359) [18]. In that study the risk was borderline significant for skin melanomas among *BRCA2* carriers (6 observed, 2.3 expected); *p* = 0.05). There was incident case of uveal melanoma in a 49 year old women with a *BRCA1* mutation in our study who was not included in the analysis.

A prior history of skin cancer was the strongest risk factor for a new skin cancer. We estimated a hazard ratio of 9.90 for a second diagnosis of melanoma after initial diagnosis in *BRCA2* mutation carriers and 11.4 for those with a *BRCA1* mutation. This is consistent with data from the general population: up to 10% of melanoma patients will go on to develop a second melanoma [21, 22]. NCCN provides recommendations for increased follow-up and screening after initial diagnosis of melanoma followed by annual skin exams with education on self-examination [23]. NCCN states that regular clinical examination has the highest diagnostic benefit and is the most cost effective method for early detection of treatable disease [23].

The small increase in melanoma risk for *BRCA1* and *BRCA2* mutation carriers is consistent with the findings of an early study of cancer risks among a cohort of women who carry *BRCA2* mutations and their first-degree relatives compared with population-specific incidence rates which found a relative risk of melanoma of 2.6; (95% CI = 1.3–5.2)^24^. Mersch et al. found an excess of a history of melanomas in a sample of mutation carriers at MD Anderson hospital [24]. Tuominen et al. also found an association with a founder mutation in a case control study of *BRCA2* in Swedish melanoma patients with an odds ratio of 2.80 (95% CI: 1.04–7.58, *p* = 0.035).[25] Debniak et al. did not find and excess of *BRCA1* or *BRCA2* mutations in a sample of melanoma patients from Poland [26]. 

In an earlier study of this cohort, we reported an excess risk of basal cell carcinoma in *BRCA1* and *BRCA2* carriers [27]. The current study expands the cohort size from 2797 women to 6207 women and extended the mean follow-up from 5.0 years to 8.1 years. To our knowledge, this is the largest prospective study addressing the risk of skin cancer in *BRCA* mutation carriers. We found the cumulative risk of all types of skin cancer reported in this cohort from age 20 to 80 years to be 14.1% for *BRCA1* mutation carriers and 10.7% for *BRCA2* mutation carriers. In comparison, the lifetime risk for basal cell carcinoma and squamous cell carcinoma in women in the general United States population has been estimated to be approximately 28–33% and 7–11% respectively for a woman born in 1994[28]. While these data suggest that the risk for skin cancer in *BRCA* mutation carriers is not increased, data on the incidence of non-melanoma skin cancer is limited as it is not included in most cancer registries [29, 30]. Our cohort has a mean birth year of 1957 which also bears on the comparison of these risks. Studies have indicated that the incidence of keratinocyte carcinoma has been steadily increasing [30].

We found that the risk for skin cancer was increased after a diagnosis of breast cancer for *BRCA1* mutation carriers (HR = 1.77, 95% CI: 1.26–2.50, *p* = 0.001) but not for *BRCA2* mutation carriers *(*HR = 1.13, 95% CI: 0.76–1.67, *p* = 0.56). A relationship between breast cancer and skin cancer has been observed in cancer registry-based studies [31–33]. A large study looking at second primary cancers after breast cancer in the general population found a similar association with SIRs for non-melanoma skin cancer and melanoma of 1.58 and 1.29 respectively [32]. 

There are a number of limitations in this study. This study of skin cancer risk was based on patient report of new cancer diagnoses in biannual questionnaires and diagnoses were not confirmed with pathology reports. Women with incident skin cancers reported either melanoma, basal cell carcinoma, or ‘skin cancer’ and it is possible that the sub-types of cancers were misclassified. It is possible that skin cancer is under-reported by study participants as well as in their first-degree relatives. We cannot estimate the risks for male *BRCA* mutation carriers. These data are derived from predominantly white women in Canada and the United States. It is possible that the risks are higher in white women in countries with greater sun exposure, such as Australia and New Zealand. We have information on current residence, but not prior residence and it is possible that early childhood exposures are relevant for skin cancer risks in adults. We do not have information on other risk factors for skin cancer such as hair color and fair skin and extent or recreational sun exposure. Our cohort was followed until a mean age of 55 years and skin cancer rates were highest above age 70. Future studies will be conducted to better estimate the lifetime cumulative rate of skin cancer among BRCA1 and BRCA2 carriers. Further, the results of this study should not be extrapolated to non-white women and there were too few non-white women in the study to conduct a robust analysis.

Prevention of skin cancer through limiting ultraviolet light (UV) exposure, use of sunscreen and early detection is important in the general population. The NCCN states that no specific screening guidelines exist for melanoma for *BRCA* mutation carriers, but that general melanoma risk management such as annual full-body skin examination and minimization of UV exposure is appropriate [34]. The results of our study are consistent with conclusions of the review article by Gumaste et al. [35] who stated there was insufficient evidence to support the claim that skin cancer is a manifestation of the *BRCA1* and *BRCA2* phenotype but recommend annual skin examinations. We suggest a referral for skin exam to a primary care provider for *BRCA* mutation carriers with a prior history of skin cancer for annual skin examination and counselling around protection from UV exposure, use of sunscreen, and self-screening to recognize the early signs of melanoma.

## Data Availability

No datasets were generated or analysed during the current study.
